# Multiple Solutions for a Class of Dirichlet Double Eigenvalue Quasilinear Elliptic Systems Involving the (*p*_1_,…, *p*_*n*_)-Laplacian Operator

**DOI:** 10.1155/2013/767989

**Published:** 2013-11-03

**Authors:** Armin Hadjian, Saleh Shakeri

**Affiliations:** Department of Mathematics, Ayatollah Amoli Branch, Islamic Azad University, P.O. Box 678, Amol, Iran

## Abstract

Existence results of three weak solutions for a Dirichlet double eigenvalue quasilinear elliptic system involving the (*p*_1_,…, *p*_*n*_)-Laplacian operator, under suitable assumptions, are established. Our main tool is based on a recent three-critical-point theorem obtained by Ricceri. We also give some examples to illustrate the obtained results.

## 1. Introduction and Preliminaries

The aim of this paper is to investigate the existence of at least three weak solutions for the following Dirichlet double eigenvalue quasilinear elliptic system:

(1) −Δpiui =λFui(x,u1,…,un)+μGui(x,u1,…,un) in  Ω,ui=0 on⁡  ∂Ω,

for 1 ≤ *i* ≤ *n*, where Δ_*p*_*i*__ *u*_*i*_ : = div⁡(|∇*u*_*i*_|^*p*_*i*_−2^∇*u*_*i*_)  is the *p*_*i*_-Laplacian operator, *Ω* ⊂ ℝ^*N*^  (*N* ≥ 1) is a nonempty bounded open set with a boundary ∂*Ω* of class *C* ^1^, *λ* and *μ* are positive parameters, and *p*_*i*_ > *N* for 1 ≤ *i* ≤ *n*. Here, *F*, *G* : *Ω* × ℝ^*n*^ → ℝ are measurable functions with respect to *x* ∈ *Ω* for every (*t*_1_,…, *t*_*n*_) ∈ ℝ^*n*^ and are *C* ^1^ with respect to (*t*_1_,…, *t*_*n*_) ∈ ℝ^*n*^ for a.e. *x* ∈ *Ω*, and *F*_*u*_*i*__ and *G*_*u*_*i*__ denote the partial derivatives of *F* and *G* with respect to *u*_*i*_, respectively.

Moreover, *F* and *G* satisfy the following additional assumptions:

(F_1_) for every *M* > 0 and every 1 ≤ *i* ≤ *n*,
  (2) $$\begin{array}{c} \underset{|(t_{1},\ldots ,t_{n})|\leq M}{\sup}\left|F_{u_{i}}\left(x,t_{1},\ldots ,t_{n}\right)\right|\in L^{1}\left(\Omega \right); \end{array}$$
(F_2_) *F*(*x*, 0,…, 0) = 0 for a.e. *x* ∈ *Ω*;
(G) for every *M* > 0 and every 1 ≤ *i* ≤ *n*,
  (3) $$\begin{array}{c} \underset{|(t_{1},\ldots ,t_{n})|\leq M}{\sup}\left|G_{u_{i}}\left(x,t_{1},\ldots ,t_{n}\right)\right|\in L^{1}\left(\Omega \right). \end{array}$$

Here and in the following, we let *X* be the Cartesian product of the *n* Sobolev spaces *W*_0_^1,*p*_*i*_^(*Ω*) for 1 ≤ *i* ≤ *n*; that is, *X* = *W*_0_^1,*p*_1_^(*Ω*) × *W*_0_^1,*p*_2_^(*Ω*) × ⋯×*W*_0_^1,*p*_*n*_^(*Ω*) equipped with the norm

(4) $$\begin{array}{c} ||u||≔\overset{n}{\underset{i=1}{\sum }}{||u_{i}||}_{p_{i}},\;u=\left(u_{1},u_{2},\ldots ,u_{n}\right), \end{array}$$

where for 1 ≤ *i* ≤ *n*,

(5) $$\begin{array}{c} {||u_{i}||}_{p_{i}}:={\left[\int_{\Omega }{\left|\nabla u_{i}\left(x\right)\right|}^{p_{i}}dx\right]}^{1/p_{i}}. \end{array}$$

Put

(6) $$\begin{array}{c} c:=\max\left\{\underset{u_{i}\in W_{0}^{1,p_{i}}\left(\Omega \right)\setminus \{0\}}{\sup}\frac{\max_{x\in \bar{\Omega }}{\left|u_{i}\left(x\right)\right|}^{p_{i}}}{{||u_{i}||}_{p_{i}}^{p_{i}}}:\text{for}\;1\leq i\leq n\right\}. \end{array}$$

Since *p*_*i*_ > *N* for 1 ≤ *i* ≤ *n*, *X* is compactly embedded in ${\left(C^{0}(\bar{\Omega })\right)}^{n}$, so that *c* < +*∞*. In addition, it is known (see [1, formula (6b)]) that

(7) sup⁡ui∈W01,pi(Ω)∖{0}max⁡x∈Ω¯|ui(x)|||ui||pi ≤N−1/piπ[Γ(1+N2)]1/N(pi−1pi−N)1−1/pi[m(Ω)]1/N−1/pi

for 1 ≤ *i* ≤ *n*, where Γ denotes the Gamma function defined by

(8) $$\begin{array}{c} \Gamma \left(t\right):=\overset{+\infty }{\underset{0}{\int }}z^{t-1}e^{-z}dz,\;\forall t>0, \end{array}$$

and *m*(*Ω*) is the Lebesgue measure of the set *Ω*, and equality occurs when *Ω* is a ball.

Moreover, let

(9) $$\begin{array}{c} D:=\underset{x\in \Omega }{\sup}\mathrm{dist}\left(x,\partial \Omega \right). \end{array}$$

Simple calculations show that there is *x* ^0^ ∈ *Ω* such that *B*(*x* ^0^, *D*)⊆*Ω*, where *B*(*x*, *r*) stands for the open ball in ℝ^*N*^ of radius *r* centered at *x*.

Put

(10) $$\begin{array}{c} \kappa_{i}:=\frac{2}{D}{\left[\frac{c\pi^{N/2}\left(D^{N}-{\left(D/2\right)}^{N}\right)\;}{\Gamma \left(1+N/2\right)\;}\right]}^{1/p_{i}} \end{array}$$

for 1 ≤ *i* ≤ *n*.

By a (weak) solution of system (1), we mean any *u* = (*u*_1_,…, *u*_*n*_) ∈ *X* such that

(11)   ∫Ω∑i=1n|∇ui(x)|pi−2∇ui(x)∇vi(x)dx −λ∫Ω∑i=1nFui(x,u1(x),…,un(x))vi(x)dx −μ∫Ω∑i=1nGui(x,u1(x),…,un(x))vi(x)dx=0

for all *v* = (*v*_1_,…, *v*_*n*_) ∈ *X*.

In the literature many papers (see, e.g., the papers [2–9] and references therein) deal with nonlinear elliptic problems. Motivated by the fact that such kind of problems is used to describe a large class of physical phenomena, many authors have studied the existence and multiplicity of solutions for (1).

The goal of this work is to establish some new criteria for system (1) to have at least three weak solutions in *X*, by means of a very recent abstract critical point result of Ricceri [10]. We first recall the following three-critical-point theorem that follows from a combination of [11, Theorem  3.6] and [10, Theorem  1].

**Lemma 1**

Let *X* be a reflexive real Banach space; let Φ : *X* → ℝ be a continuously Gâteaux differentiable and sequentially weakly lower semicontinuous functional whose Gâteaux derivative admits a continuous inverse on X*, bounded on bounded subsets of *X*; Ψ : *X* → ℝ a continuously Gâteaux differentiable functional whose Gâteaux derivative is compact such that

(12) $$\begin{array}{c} \Phi \left(0\right)=\Psi \left(0\right)=0. \end{array}$$

Assume that there exists *r* > 0 and $\bar{x}\in X$, with $r<\Phi (\bar{x})$, such that

(a_1_) $\left(\sup_{\Phi (x)\leq r}\Psi (x)/r\right)<\Psi (\bar{x})/\Phi (\bar{x})$;
(a_2_) for each$\;\lambda \in \Lambda_{r}:=]\Phi (\bar{x})/\Psi (\bar{x}),r/\sup_{\Phi (x)\leq r}\Psi (x)[$, the functional Φ − *λ*Ψ is coercive.

Then, for each compact interval [*a*, *b*]⊆Λ_*r*_, there exists *ρ* > 0 with the following property: for every *λ* ∈ [*a*, *b*] and every *C* ^1^ functional Γ : *X* → ℝ with compact derivative, there exists *δ* > 0 such that, for each *μ* ∈ [0, *δ*], the equation

(13) $$\begin{array}{c} \Phi^{\prime }\left(x\right)-\lambda \Psi^{\prime }\left(x\right)-\mu \Gamma^{\prime }\left(x\right)=0 \end{array}$$

has at least three solutions in *X* whose norms are less than *ρ*.

For other basic notations and definitions, we refer the reader to [12–14].

## 2. Main Result

In the present section we discuss the existence of multiple solutions for system (1). For any *γ* > 0, we denote by *K*(*γ*) the set

(14) $$\begin{array}{c} \left\{\left(t_{1},\ldots ,t_{n}\right)\in {\mathbb{R}}^{n}:\overset{n}{\underset{i=1}{\sum }}\frac{{\left|t_{i}\right|}^{p_{i}}}{p_{i}}\leq \gamma \right\}. \end{array}$$

This set will be used in some of our hypotheses with appropriate choices of *γ*.

We formulate our main result as follows.

**Theorem 2**

Assume that there exist two positive constants *θ* and *δ* with ∑_*i*=1_^*n*^((*δκ*_*i*_)^*p*_*i*_^/*p*_*i*_) > (*θ*/∏_*i*=1_^*n*^ *p*_*i*_) such that

(b_1_) *F*(*x*, *t*_1_,…, *t*_*n*_) ≥ 0 for a.e. *x* ∈ *Ω*∖*B*(*x* ^0^, *D*/2) and all *t*_*i*_ ∈ [0, *δ*] for 1 ≤ *i* ≤ *n*;
(b_2_) (15) θ∏i=1npi∫B(x0,D/2)F(x,δ,…,δ)dx −∑i=1n(δκi)pipi∫Ωsup⁡(t1,…,tn)∈K(θ/∏i=1npi)  F(x,t1,…,tn)dx>0;
(b_3_) (16) limsup⁡(|t1|,…,|tn|)→(+∞,…,+∞)F(x,t1,…,tn)∑i=1n(|ti|pi/pi)   <∏i=1npim(Ω)θ∫Ωsup⁡(t1,…,tn)∈K(θ/∏i=1npi)  F(x,t1,…,tn)dx,

uniformly with respect to *x* ∈ *Ω*.

Then, setting

(17) $$\begin{array}{c} \Lambda :=]\frac{\sum_{i=1}^{n}\left({\left(\delta \kappa_{i}\right)}^{p_{i}}/p_{i}\right)}{c\int_{B(x^{0},D/2)\;}F\left(x,\delta ,\ldots ,\delta \right)dx},\frac{\theta }{\left(c\prod_{i=1}^{n}p_{i}\right)\int_{\Omega }\sup_{\left(t_{1},\ldots ,t_{n})\in K(\theta /\prod_{i=1}^{n}p_{i}\right)}F\left(x,t_{1},\ldots ,t_{n}\right)dx}[, \end{array}$$

for each compact interval [*a*, *b*]⊆Λ, there exists *ρ* > 0 with the following property: for every *λ* ∈ [*a*, *b*], there exists *δ* > 0 such that, for each *μ* ∈ [0, *δ*], system (1) admits at least three weak solutions in *X* whose norms are less than *ρ*.

**Proof**

Our aim is to apply Lemma 1 to our problem. To this end, for each *u* = (*u*_1_,…, *u*_*n*_) ∈ *X*, we let the functionals Φ, Ψ : *X* → ℝ be defined by

(18) $$\begin{array}{c} \Phi \left(u\right):=\overset{n}{\underset{i=1}{\sum }}\frac{{||u_{i}||}_{p_{i}}^{p_{i}}}{p_{i}},\\ \Psi \left(u\right):=\int_{\Omega }F\left(x,u_{1}\left(x\right),\ldots ,u_{n}\left(x\right)\right)dx. \end{array}$$

Clearly, Φ is bounded on each bounded subset of *X* and it is known that Φ and Ψ are well-defined and continuously Gâteaux differentiable functionals whose derivatives at the point *u* = (*u*_1_,…, *u*_*n*_) ∈ *X* are the functionals Φ′(*u*) and Ψ′(*u*) given by

(19) $$\begin{array}{c} \Phi^{\prime }\left(u\right)\left(v\right)=\int_{\Omega }\overset{n}{\underset{i=1}{\sum }}{\left|\nabla u_{i}\left(x\right)\right|}^{p_{i}-2}\nabla u_{i}\left(x\right)\nabla v_{i}\left(x\right)dx,\\ \Psi^{\prime }\left(u\right)\left(v\right)=\int_{\Omega }\overset{n}{\underset{i=1}{\sum }}F_{u_{i}}\left(x,u_{1}\left(x\right),\ldots ,u_{n}\left(x\right)\right)v_{i}\left(x\right)dx \end{array}$$

for every *v* = (*v*_1_,…, *v*_*n*_) ∈ *X*, and Φ is sequentially weakly lower semicontinuous (see Proposition  25.20 of [14]). Also, Φ′ : *X* → X* is a uniformly monotone operator in *X* (for more details, see (2.2) of [15]), and since Φ′ is coercive and hemicontinuous in *X*, by applying Minty-Browder theorem (Theorem  26.A of [14]), Φ′ admits a continuous inverse on X*.

We claim that Ψ′ : *X* → X* is a compact operator. To this end, it is enough to show that Ψ′ is strongly continuous on *X*. For this, for fixed (*u*_1_,…, *u*_*n*_) ∈ *X*, let  (*u*_1*m*_,…, *u*_*nm*_)→(*u*_1_,…, *u*_*n*_) weakly in *X* as *m* → +*∞*. Then we have that (*u*_1*m*_,…, *u*_*nm*_) converges uniformly to (*u*_1_,…, *u*_*n*_) on *Ω* as *m* → +*∞* (see [14]). Since *F*(*x*, ·,…, ·) is *C* ^1^ in ℝ^*n*^ for every *x* ∈ *Ω*, the derivatives of *F* are continuous in ℝ^*n*^ for every *x* ∈ *Ω*, so fo 1 ≤ *i* ≤ *n*, *F*_*u*_*i*__(*x*, *u*_1*m*_,…, *u*_*nm*_) → *F*_*u*_*i*__(*x*, *u*_1_,…, *u*_*n*_) strongly as *m* → +*∞*. By the Lebesgue control convergence theorem, Ψ′(*u*_1*m*_,…, *u*_*nm*_) → Ψ′(*u*_1_,…, *u*_*n*_) strongly as *m* → +*∞*. Thus we proved that Ψ′ is strongly continuous on *X*, which implies that Ψ′ is a compact operator by [14, Proposition  26.2]. Hence the claim is true.

Moreover, we have

(20) $$\begin{array}{c} \Phi \left(0\right)=\Psi \left(0\right)=0. \end{array}$$

Next, put *w*(*x*) = (*w*_1_(*x*),…, *w*_*n*_(*x*)) such that for 1 ≤ *i* ≤ *n*,

(21) wi(x):={0x∈Ω∖B(x0,D),2δD(D−∑j=1N(xj−xj0)2)  x∈B(x0,D)∖B(x0,D/2),δx∈B(x0,D/2),

and *r* : = (*θ*/*c*∏_*i*=1_^*n*^ *p*_*i*_). Clearly *w* = (*w*_1_,…, *w*_*n*_) ∈ *X* and, in particular, one has for 1 ≤ *i* ≤ *n*,

(22) $$\begin{array}{c} {||w_{i}||}_{p_{i}}^{p_{i}}=\frac{\pi^{N/2}}{\Gamma \left(1+N/2\right)\;}\left(D^{N}-{\left(D/2\right)}^{N}\right){\left(\frac{2\delta }{D}\right)}^{p_{i}}. \end{array}$$

So

(23) $$\begin{array}{c} \Phi \left(w\right)=\frac{\pi^{N/2}}{\Gamma \left(1+N/2\right)}\left(D^{N}-{\left(D/2\right)}^{N}\right)\overset{n}{\underset{i=1}{\sum }}\frac{1}{p_{i}}{\left(\frac{2\delta }{D}\right)}^{p_{i}}. \end{array}$$

Bearing in mind that ∑_*i*=1_^*n*^((*δκ*_*i*_)^*p*_*i*_^/*p*_*i*_) > (*θ*/∏_*i*=1_^*n*^ *p*_*i*_) and that ||*w*_*i*_||_*p*_*i*__^*p*_*i*_^ = ((*δκ*_*i*_)^*p*_*i*_^/*c*) for 1 ≤ *i* ≤ *n*, one has Φ(*w*) > *r*.

Since 0 ≤ *w*_*i*_(*x*) ≤ *δ* for each *x* ∈ *Ω* for 1 ≤ *i* ≤ *n*, condition (b_1_) ensures that

(24) ∫Ω∖B(x0,D)F(x,w1(x),…,wn(x))dx +∫B(x0,D)∖B(x0,D/2)  F(x,w1(x),…,wn(x))dx≥0.

Hence

(25) ∫ΩF(x,w1(x),…,wn(x))dx  ≥∫B(x0,D/2)F(x,δ,…,δ)dx.

Moreover, owing to assumption (b_2_), we have

(26) ∫Ωsup⁡(t1,…,tn)∈K(θ/∏i=1npi)F(x,t1,…,tn)dx<θ(∑i=1n((δκi)pi/pi))(∏i=1npi)∫B(x0,D/2)F(x,δ,…,δ)dx≤θc∫ΩF(x,w1(x),…,wn(x))dx∑i=1n(∏j=1j≠inpj)||wi||pipi  .

Taking into account that for each *u*_*i*_ ∈ *W*_0_^1,*p*_*i*_^(*Ω*)  

(27) $$\begin{array}{c} \underset{x\in \Omega }{\sup}{\left|u_{i}\left(x\right)\right|}^{p_{i}}\leq c{||u_{i}||}_{p_{i}}^{p_{i}} \end{array}$$

for 1 ≤ *i* ≤ *n* (see (6)), we have that

(28) $$\begin{array}{c} \underset{x\in \Omega }{\sup}\overset{n}{\underset{i=1}{\sum }}\frac{{\left|u_{i}\left(x\right)\right|}^{p_{i}}}{p_{i}}\leq c\overset{n}{\underset{i=1}{\sum }}\frac{{||u_{i}||}_{p_{i}}^{p_{i}}}{p_{i}}=c\Phi \left(u\right) \end{array}$$

for every *u* = (*u*_1_,…, *u*_*n*_) ∈ *X*, and taking into account (26) and (28), it follows that

(29) sup⁡u∈Φ−1(]−∞,r])Ψ(u)   =sup⁡Φ(u)≤r∫ΩF(x,u1(x),…,un(x))dx   ≤∫Ωsup⁡(t1,…,tn)∈K(θ/∏i=1npi)F(x,t1,…,tn)dx   <θc∫ΩF(x,w1(x),…,wn(x))dx∑i=1n(∏j=1j≠inpj)||wi||pipi   =θc∏i=1npi∫ΩF(x,w1(x),…,wn(x))dx∑i=1n(||wi||pipi/pi)   =rΨ(w)Φ(w).

Therefore, assumption (a_1_) of Lemma 1 is satisfied.

Now, fixed *λ* ∈ Λ, due to (b_3_), there exist two constants *γ*, *ϑ* ∈ ℝ with

(30) $$\begin{array}{c} 0<\gamma <\frac{\prod_{i=1}^{n}p_{i}}{m\left(\Omega \right)\theta }\int_{\Omega }\underset{\left(t_{1},\ldots ,t_{n})\in K(\theta /\overset{n}{\underset{i=1}{\prod }}p_{i}\right)}{\sup}F\left(x,t_{1},\ldots ,t_{n}\right)dx \end{array}$$

such that

(31) $$\begin{array}{c} F\left(x,t_{1},\ldots ,t_{n}\right)\leq \gamma \left(\overset{n}{\underset{i=1}{\sum }}\frac{{\left|t_{i}\right|}^{p_{i}}}{p_{i}}\right)+\vartheta \end{array}$$

for all *x* ∈ *Ω* and for all (*t*_1_,…, *t*_*n*_) ∈ ℝ^*n*^. Fix *u* = (*u*_1_,…, *u*_*n*_) ∈ *X*. Then

(32) $$\begin{array}{c} F\left(x,u_{1}\left(x\right),\ldots ,u_{n}\left(x\right)\right)\leq \gamma \left(\overset{n}{\underset{i=1}{\sum }}\frac{{\left|u_{i}\left(x\right)\right|}^{p_{i}}}{p_{i}}\right)+\vartheta \end{array}$$

for all *x* ∈ *Ω*. So, for any fixed *λ* ∈ Λ, from (28) and (32), we have

(33) Φ(u)−λΨ(u) =∑i=1n||ui||pipipi−λ∫ΩF(x,u1(x),…,un(x))dx ≥∑i=1n||ui||pipipi−λγ(∫Ω∑i=1n|ui(x)|pipidx)−λϑm(Ω) ≥∑i=1n||ui||pipipi−λγ(cm(Ω)∑i=1n||ui||pipipi)−λϑm(Ω) ≥(1−m(Ω)γθ(∏i=1npi)∫Ωsup⁡(t1,…,tn)∈K(θ/∏i=1npi)F(x,t1,…,tn)dx)  ×∑i=1n||ui||pipipi  −m(Ω)ϑθ(c∏i=1npi)∫Ωsup⁡(t1,…,tn)∈K(θ/∏i=1npi)F(x,t1,…,tn)dx,

and thus

(34) $$\begin{array}{c} \underset{||u||\to +\infty }{\lim}\left(\Phi \left(u\right)-\lambda \Psi \left(u\right)\right)=+\infty , \end{array}$$

which means that the functional Φ − *λ*Ψ is coercive. Then, also condition (a_2_) of Lemma 1 holds.

In addition, since *G* : *Ω* × ℝ^*n*^ → ℝ is a measurable function with respect to *x* ∈ *Ω* for every (*t*_1_,…, *t*_*n*_) ∈ ℝ^*n*^ and is *C* ^1^ with respect to (*t*_1_,…, *t*_*n*_) ∈ ℝ^*n*^ for a.e. *x* ∈ *Ω*, satisfying condition (G), the functional

(35) $$\begin{array}{c} \Gamma \left(u\right)=\int_{\Omega }G\left(x,u_{1}\left(x\right),\ldots ,u_{n}\left(x\right)\right)dx \end{array}$$

is well defined and continuously Gâteaux differentiable on *X* with a compact derivative, and

(36) $$\begin{array}{c} \Gamma^{\prime }\left(u\right)\left(v\right)=\int_{\Omega }\overset{n}{\underset{i=1}{\sum }}G_{u_{i}}\left(x,u_{1}\left(x\right),\ldots ,u_{n}\left(x\right)\right)v_{i}\left(x\right)dx \end{array}$$

for all *u* = (*u*_1_,…, *u*_*n*_), *v* = (*v*_1_,…, *v*_*n*_) ∈ *X*. Thus, all the hypotheses of Lemma 1 are satisfied. Also note that the solutions of the equation

(37) $$\begin{array}{c} \Phi^{\prime }\left(u\right)-\lambda \Psi^{\prime }\left(u\right)-\mu \Gamma^{\prime }\left(u\right)=0 \end{array}$$

are exactly the weak solutions of (1). So, the conclusion follows from Lemma 1.

We now point out the following special case of Theorem 2 when *F* does not depend on *x* ∈ *Ω*.

**Theorem 3**

Let *F* : ℝ^*n*^ → ℝ be a *C* ^1^-function and assume that there exist two positive constants *θ* and *δ* with ∑_*i*=1_^*n*^((*δκ*_*i*_)^*p*_*i*_^/*p*_*i*_) > (*θ*/∏_*i*=1_^*n*^ *p*_*i*_) such that

(b_4_) *F*(*t*_1_,…, *t*_*n*_) ≥ 0 for all *t*_*i*_ ∈ [0, *δ*] for 1 ≤ *i* ≤ *n*;
(b_5_) (38) θπN/2Γ(1+(N/2))∏i=1npi(D2)NF(δ,…,δ) −m(Ω)∑i=1n(δκi)pipisup⁡(t1,…,tn)∈K(θ/∏i=1npi)F(t1,…,tn)>0;
(b_6_) limsup⁡_(|*t*_1_|,…,|*t*_*n*_|)→(+*∞*,…,+*∞*)_ *F*(*t*_1_,…, *t*_*n*_)/∑_*i*=1_^*n*^(|*t*_*i*_|^*p*_*i*_^/*p*_*i*_) ≤ 0.  

Then, setting

(39) $$\begin{array}{c} \Lambda :=]\frac{\Gamma \left(1+N/2\right)\;\sum_{i=1}^{n}\left({\left(\delta \kappa_{i}\right)}^{p_{i}}/p_{i}\right)}{c\pi^{N/2}F\left(\delta ,\ldots ,\delta \right)\;}{\left(\frac{2}{D}\right)}^{N},\frac{\theta }{\left(c\prod_{i=1}^{n}p_{i}\right)m\left(\Omega \right)\sup_{\left(t_{1},\ldots ,t_{n})\in K(\theta /\prod_{i=1}^{n}p_{i}\right)}F\left(t_{1},\ldots ,t_{n}\right)\;}[, \end{array}$$

for each compact interval [*a*, *b*]⊆Λ, there exists *ρ* > 0 with the following property: for every *λ* ∈ [*a*, *b*], there exists *δ* > 0 such that, for each *μ* ∈ [0, *δ*], the system

(40) −Δpiui=λFui(u1,…,un)+μGui(x,u1,…,un) in  Ω,ui=0 on  ∂Ω,

for 1 ≤ *i* ≤ *n*, admits at least three weak solutions in *X* whose norms are less than *ρ*.

**Proof**

Set *F*(*x*, *t*_1_,…, *t*_*n*_) = *F*(*t*_1_,…, *t*_*n*_) for all *x* ∈ *Ω* and *t*_*i*_ ∈ ℝ for 1 ≤ *i* ≤ *n*. Since ∫_*B*(*x*^0^,*D*/2)_ *F*(*δ*,…, *δ*)*dx* = (*π* ^*N*/2^/Γ(1 + *N*/2))(*D*/2)^*N*^ *F*(*δ*,…, *δ*), Theorem 2 ensures the conclusion.

Let *κ* = *κ*_1_ and *p* = *p*_1_. Then we have the following existence result.

**Corollary 4**

Let *f* : ℝ → ℝ be a continuous function and let *g* : *Ω* × ℝ → ℝ be an *L* ^1^-Carathéodory function. Put *F*(*t*) = ∫_0_^*t*^ *f*(*ξ*)*dξ* for each *t* ∈ ℝ and assume that there exist two positive constants *θ* and *δ* with (*δκ*)^*p*^ > *θ* such that

(b_7_) *F*(*t*) ≥ 0 for all *t* ∈ [0, *δ*];  
(b_8_) $\left(\theta \pi^{N/2}/\Gamma (1+N/2)\right){\left(D/2\right)}^{N}F(\delta )-m(\Omega ){\left(\delta \kappa \right)}^{p}\sup_{t\in [-\sqrt[{p}]{\theta },\sqrt[{p}]{\theta }]}F(t)>0$;
(b_9_) limsup⁡_|*t*|→+*∞*_ *F*(*t*)/|*t*|^*p*^ ≤ 0.  

Then, setting

(41) Λ:=]Γ(1+N/2)(δκ)p(pc)πN/2F(δ)  (2D)N,θm(Ω)(pc)sup⁡t∈[−θp,θp]F(t)  [,

for each compact interval [*a*, *b*]⊆Λ, there exists *ρ* > 0 with the following property: for every *λ* ∈ [*a*, *b*], there exists *δ* > 0 such that, for each *μ* ∈ [0, *δ*], the problem

(42) $$\begin{array}{c} -\Delta_{p}u=\lambda f\left(u\right)+\mu g\left(x,u\right)\;in\;\Omega ,\\ u=0\;on\;\partial \Omega \end{array}$$

admits at least three weak solutions in *W*_0_^1,*p*^(*Ω*) whose norms are less than *ρ*.

Now, we want to point out a simple consequence of Corollary 4 in the case when *N* = 1 and *p* = 2. For simplicity, we fix *Ω* = (*α*, *β*) and note that in this situation, *c* = (*β* − *α*)/4 and *κ* = ((*β* − *α*)/*D*)^1/2^.

**Corollary 5**

Let *f* : ℝ → ℝ be a continuous function and *g* : (*α*, *β*) × ℝ → ℝ an *L* ^1^-Carathéodory function. Put *F*(*t*) = ∫_0_^*t*^ *f*(*ξ*)*dξ* for each *t* ∈ ℝ and assume that there exist two positive constants *θ* and *δ* with (*δ* ^2^(*β* − *α*)/*D*) > *θ* such that assumption (b_7_) in Corollary 4 holds, and

(b_10_) $(\theta D)F(\delta )-\left(\delta^{2}{\left(\beta -\alpha \right)}^{2}/D\right)\sup_{t\in [-\sqrt{\theta },\sqrt{\theta }]}F\left(t\right)>0$;
(b_11_) limsup⁡_|*t*|→+*∞*_(*F*(*t*)/*t* ^2^) ≤ 0.  

Then, setting

(43) Λ:=]2δ2D2F(δ),2θ(β−α)2sup⁡t∈[−θ,θ]F(t)[,

for each compact interval [*a*, *b*]⊆Λ, there exists *ρ* > 0 with the following property: for every *λ* ∈ [*a*, *b*], there exists *δ* > 0 such that, for each *μ* ∈ [0, *δ*], the problem

(44) $$\begin{array}{c} {-u}^{\prime \prime }=\lambda f\left(u\right)+\mu g\left(x,u\right)\;in\;\left(\alpha ,\beta \right),\\ u\left(\alpha \right)=u\left(\beta \right)=0 \end{array}$$

admits at least three classical solutions in *C* ^2^([*α*, *β*]) whose norms are less than *ρ*.

## 3. Examples

 First, we present an example of the application of Theorem 3.

**Example 1**

Let *Ω* = {(*x*, *y*) ∈ ℝ^2^ : *x* ^2^ + *y* ^2^ < 4}. Consider the system

(45) −Δ3u1=λe1−u1u111(12−u1)+μGu1(x,y,u1,u2) in   Ω,−Δ3u2=λe1−u2u213(14−u2)+μGu2(x,y,u1,u2) in   Ω,u1=u2=0 on⁡  ∂Ω,

where *G* : *Ω* × ℝ^2^ → ℝ is an arbitrary function which is measurable with respect to (*x*, *y*) ∈ *Ω* for every (*t*_1_, *t*_2_) ∈ ℝ^2^ and is *C* ^1^ with respect to (*t*_1_, *t*_2_) ∈ ℝ^2^ for a.e. (*x*, *y*) ∈ *Ω*, satisfying

(46) $$\begin{array}{c} \underset{|(t_{1},t_{2})|\leq M}{\sup}\left|G_{u_{i}}\left(x,y,t_{1},t_{2}\right)\right|\in L^{1}\left(\Omega \right) \end{array}$$

for every *M* > 0 and *i* = 1,2. Taking into account *c* = 4/*π*, picking *x* ^0^ = (0,0) and

(47) $$\begin{array}{c} F\left(t_{1},t_{2}\right)=e^{1-t_{1}}t_{1}^{12}+e^{1-t_{2}}t_{2}^{14} \end{array}$$

for each (*t*_1_, *t*_2_) ∈ ℝ^2^, so that *D* = 2 and $\kappa_{1}=\kappa_{2}=\sqrt[{3}]{12}$, by choosing *δ* = *θ* = 3, we have *F*(*t*_1_, *t*_2_) ≥ 0 for all (*t*_1_, *t*_2_)∈[0,3]×[0,3] and

(48) $$\begin{array}{c} \underset{\left(|t_{1}|,|t_{2}|)\to (+\infty ,+\infty \right)}{\mathrm{limsup}}\frac{F\left(t_{1},t_{2}\right)}{\left(1/3\right){\left|t_{1}\right|}^{3}+\left(1/3\right){\left|t_{2}\right|}^{3}}=0. \end{array}$$

We see that

(49) sup⁡|t1|3+|t2|3≤1(e1−t1t112+e1−t2t214)     ≤sup⁡|t1|≤1e1−t1t112+sup⁡|t2|≤1e1−t2t214=2e2,

which gives that

(50) θπp1p2(D2)2F(δ,δ)  −m(Ω)∑i=12(δκi)pipisup⁡(t1,t2)∈K(θ/p1p2)  F(t1,t2) ≥π3F(3,3)−(4π)(23·33)  ×(sup⁡|t1|≤1e1−t1t112+sup⁡|t2|≤1e1−t2t214) =311·10πe2−(26·33·πe2)>0.

Hence, all the assumptions of Theorem 3, with *p*_1_ = *p*_2_ = 3, are satisfied. So, setting

(51) $$\begin{array}{c} \Lambda =]\frac{e^{2}}{3^{9}\cdot 5},\frac{1}{2^{5}\cdot 3e^{2}}[, \end{array}$$

for each compact interval [*a*, *b*]⊆Λ, there exists *ρ* > 0 with the following property: for every *λ* ∈ [*a*, *b*], there exists *δ* > 0 such that, for each *μ* ∈ [0, *δ*], system (45) has at least three weak solutions in *W*_0_^1,3^(*Ω*) × *W*_0_^1,3^(*Ω*) whose norms are less than *ρ*.

The next example follows directly by Corollary 5.

**Example 2**

Suppose *f*(*t*) = (1/10)*t* ^9^ *e* ^−*t*^(10 − *t*) for all *t* ∈ ℝ. Then one has *F*(*t*) = (1/10)*t* ^10^ *e* ^−*t*^ for all *t* ∈ ℝ. Now, consider the following two-point boundary value problem

(52) $$\begin{array}{c} -u^{\prime \prime }=\lambda f\left(u\right)+\mu g\left(x,u\right)\;\text{in}\;\left(-1,1\right),\\ u\left(-1\right)=u\left(1\right)=0, \end{array}$$

where *g* : (−1,1) × ℝ → ℝ is an arbitrary *L* ^1^-Carathéodory function. Note that, in this case, *x* ^0^ = 0, *D* = 1, *c* = 1/2, and $\kappa =\sqrt{2}$. In fact, by choosing *θ* = 1 and *δ* = 2, we have *F*(*t*) ≥ 0 for all *t* ∈ [0,2]. Also

(53) (θD)F(δ)−δ2(β−α)2Dsup⁡t∈[−θ,θ]F(t) =F(2)−16F(−1) =15(29e−2−8e) >0,

which shows that assumption (b_10_) is fulfilled. Furthermore, we have limsup⁡_|*t*|→+*∞*_(*F*(*t*)/*t* ^2^) = 0. Thus, all hypotheses of Corollary 5 are satisfied. So, setting Λ = ](5*e* ^2^/2^7^), (5/*e*)[, for each compact interval [*a*, *b*]⊆Λ, there exists *ρ* > 0 with the following property: for every *λ* ∈ [*a*, *b*], there exists *δ* > 0 such that, for each *μ* ∈ [0, *δ*], problem (52) has at least three classical solutions in *C* ^2^([−1,1]) whose norms are less than *ρ*.

In particular, there exist two positive constants *ρ* and *δ* such that, for each *μ* ∈ [0, *δ*], the problem

(54) $$\begin{array}{c} -u^{\prime \prime }=f\left(u\right)+\mu g\left(x,u\right)\;\text{in}\;\left(-1,1\right),\\ u\left(-1\right)=u\left(1\right)=0 \end{array}$$

admits at least three classical solutions in *C* ^2^([−1,1]) whose norms are less than *ρ*.
